# Consequences of Pathogen Lists: Why Some Diseases May Continue to Plague Us

**DOI:** 10.4269/ajtmh.18-0801

**Published:** 2019-01-14

**Authors:** David M. Brett-Major, Trina Racine, Gary P. Kobinger

**Affiliations:** 1 Department of Preventive Medicine and Biostatistics, F. Edward Hébert School of Medicine, Uniformed Services University, Bethesda, Maryland;; 2 Department of Medical Microbiology, University of Manitoba, Winnipeg, Canada;; 3 Centre de Recherche en Infectiologie, Centre Hospitalier Universitaire de Québec, Université Laval, Québec City, Canada;; 4 Department of Immunology, University of Manitoba, Winnipeg, Canada;; 5 Department of Pathology and Laboratory Medicine, School of Medicine, University of Pennsylvania, Philadelphia, Pennsylvania

## Abstract

The current strategy used by many funding agencies for determining how money is spent on research to help prevent infectious disease outbreaks is based on pathogen-specific priority lists. Listing disease threats provides focus for business and research planning conducive to specific goals of developing a drug, or a vaccine, or other particular product. But, this singular type of focus has consequences. This perspective explores the consequences of lists, and describes how parallel programming independent of disease lists that address what we need to do to prevent and mitigate emerging disease risks may provide benefits out of reach of a singular focus on what products we need to have.

## How do funding choices help advance research against threats before they become major epidemics? How has our love for listing diseases prepared us against future outbreaks? Maybe, we should do lists completely differently.

Global outbreak preparedness efforts increase and mature in fits and spurts. Global recognition of threats such as the Severe Acute Respiratory Syndrome coronavirus (SARS-CoV) and avian influenza A (H5N1) in the early 2000s pushed alert, outbreak, and response mechanisms, including a revision of the International Health Regulations at the World Health Organization (WHO). Eventually, in concert with country-level initiatives, organizing forces started concentrating more on research related to outbreaks, too. In 2013, world attention was focused on the pandemic potential of Middle East respiratory syndrome coronavirus (MERS-CoV) and avian influenza A (H7N9), only to be surprised by the 2014–2016 Ebola virus disease epidemic in West Africa. However, the large scale of the Ebola epidemic and particular difficulties in trying to do research under stress in places where constructive traditions of research were not already in place highlighted the need for a more proactive approach. As a result, the WHO research and development (R&D) Blueprint was born. (http://www.who.int/blueprint/en/)

Where are we today in being ready for pandemic threats? Since 2014, we have had two major Ebola virus disease outbreaks in the Democratic Republic of Congo—one still ongoing—and a Marburg disease outbreak in Uganda. Yet, there remains no licensed therapeutic medication for either pathogen. There was also a large plague outbreak in Madagascar that persisted despite the existence of a simple, known, therapeutic agent. What, then, are we doing wrong?

In this piece, we attack lists of diseases and use a WHO list as an example for discussion. We make a case that although convenient for business and research governance, and probably necessary as part of a more comprehensive approach, such lists presently dominate thinking around R&D funding and as such result in unsatisfactory consequences to achieving patient- and community-centered outcomes. Making a list of anything is a reductive process. It requires the smashing together of many interests and suppositions about what is true and necessary. Such lists sometimes are defended with the idea that lists provide broad clarity. This carries two flawed presumptions—first, that transparency results and, second, that diverse stakeholders can be treated equally. To the first flaw, very few disease list makers disclose how their lists are generated, let alone share those lists explicitly. The WHO and the Coalition for Epidemic Preparedness Innovations (CEPI) do, in part because resource mobilization is part of their mandate. The National Institutes for Health (NIH) do so inadvertently in retrospect, through mandatory public disclosure of what has been spent, but this does not reveal their pre-expenditure strategy. Most other funders, private foundations and governmental entities alike, simply do not share anything about their lists and communicate solely through calls for proposals that vary in how much they disclose regarding spending strategy. To the second flaw, disease lists have a glaring original sin. Lists implicitly communicate what is most important to their creator, and to date, organizations creating lists think individual viral diseases are most important. We argue that although useful to the extent of aiding in the development of specific tools, this is thinking done in the wrong direction.

The WHO R&D Blueprint is a strategy and preparedness plan that develops research agendas on a predetermined list of priority pathogens not otherwise covered by major programs. These are infectious disease-causing agents that a panel of experts believes are most likely to cause a health emergency and for which there are insufficient effective countermeasures. They select them through a process comparing individual disease threats against each other for characteristics relevant to causing pandemics and so harm, together with whether tools are available to fight them. What most of the world sees from this R&D Blueprint process is arguably its smallest product, the list. The list receives media attention and is used by external funding entities. Most notably, CEPI has used the list as a starting point for its endeavors. The CEPI is a multi-stakeholder funding entity formed for the purpose of bringing vaccine and related products against potential epidemic threats that are close to licensure to completion.

Understanding a list’s purpose is key to knowing what to do with a list. In general, we love lists. Managing public resources is a heady responsibility. Priorities must be set. Another notable list is the U.S. Federal Select Agent Program’s list. This list governs regulatory practice when handling infectious disease-causing agents or their products. Although built around laboratory and specimen handling requirements, it is used as a guidepost for the generation of a subset of lists that drive advanced development of medical countermeasures meant for stockpiles, such as that elected by the U.S. Government’s Biomedical Advanced Research and Development Authority (BARDA).

### These lists are different, built for different purposes, and yet often are read and compared in the same way. Worse, the lists look alike.

They focus on the disease-causing pathogens, specifically. Infectious disease research portfolios are organized similarly. A consequence of this is that we miss opportunities to seek broadly applicable solutions to challenges in preventing and mitigating epidemics.

## What if we did something completely different?

What if we instead focused on challenges posed by multiple pathogens that need to be overcome to prevent and mitigate an epidemic? Instead of saying, we do not have a vaccine against SARS-CoV, so we should list SARS-CoV, we say, we need a way to rapidly make effective vaccines and get them ready for use in humans quickly. Examples of efforts could include technologies that rapidly find vaccine targets; safe, insertable technologies for delivering an immunogenic protein and those that quickly and accurately assess toxicity and immune performance of candidate products before placing them in people; and, just as importantly, improving the regulatory pathways that these data feed.

**Figure 1.**
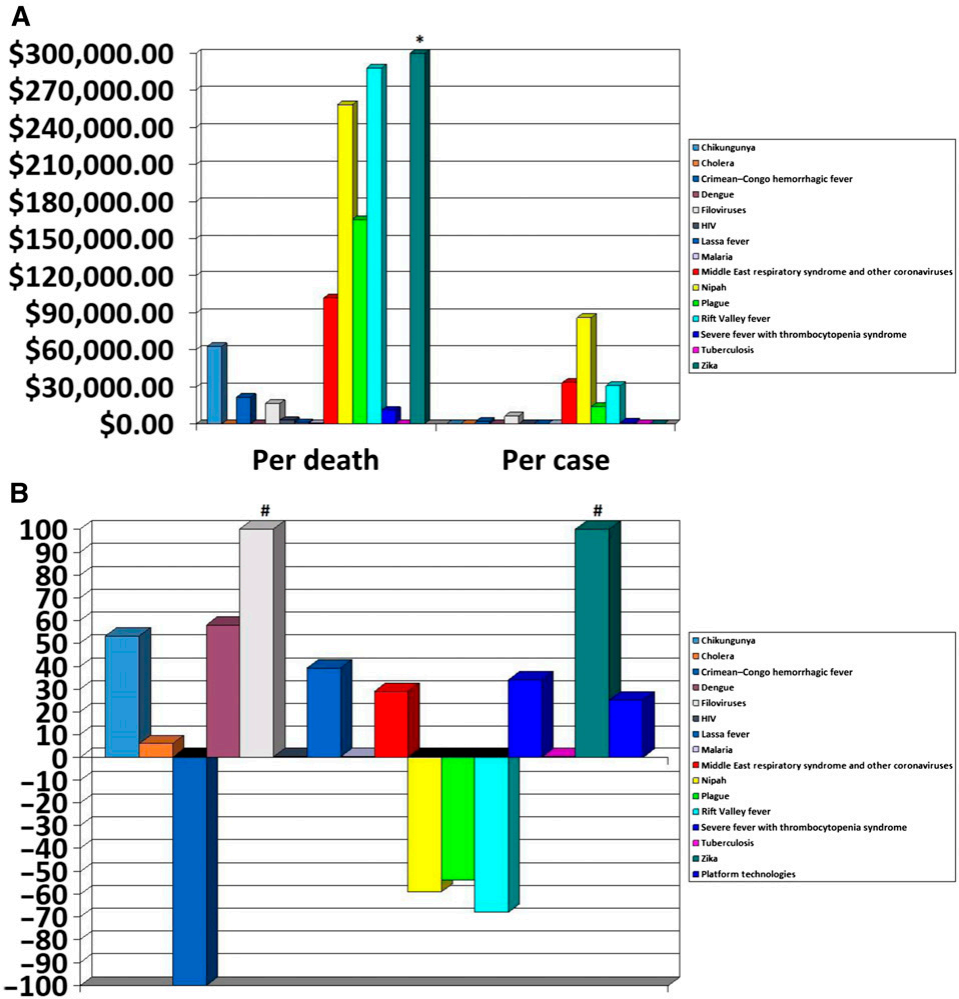
Recent National Institutes for Health (NIH) emerging disease funding. (**A**) Recent NIH investment against case and death burdens by disease pathogen. Employing multiple open-source estimates for average annual case and death burdens, and a normalized 5-year annual investment when available, or 2013/2016 average when 5-year data are not available from https://report.nih.gov/categorical_spending.aspx, published July 3, 2017 (new summary data through 2017 but not detailed information were published in May 2018, so have not been incorporated); (**B**) Percent changes in individual emerging disease investments comparing 2013 and 2016; *indicates that the investment amount exceeds scale; for Zika, normalized annual investment per death was approximately USD 4.4M; #percent change is greater than scale: for Filoviruses, 640% increase and for Zika, the increase went from 0 to more than 61M USD. This figure appears in color at www.ajtmh.org.

We explored NIH-reported research funding awards in the context of known or estimated disease burden focusing on diseases that are discussed in the R&D Blueprint and adding comparators (Figure 1). We focused on NIH funding for its breadth and transparency to illustrate the issues, although the figure does not incorporate other funders in this space, such as CEPI, the Gates Foundation, BARDA, the Wellcome Trust, and other U.S., international, and foreign donors, and may have missed some NIH platform investments because of the limitations of the data’s taxonomy. Small changes in case burden for less common diseases make marked differences in comparative costs in the figure. Also, investments that attempt to curtail future case burden are different from reactive funding against existing high case burdens, such as with Zika virus. However, it provides some context for scale and comparison of investment, and illustrates recent shifts in funding. Of note, in Figure 1B, Crimean–Congo hemorrhagic fever (CCHF), Nipah, and Rift Valley fever all experienced large decreases in NIH funding between 2013 and 2016, despite being flagged by the WHO priority lists in 2015 and 2016. National Institutes for Health consistently spends the largest single allocations of money on the major infectious disease killers, HIV/AIDS, malaria, and tuberculosis (TB); they are included in the figure as a frame of reference for scale when looking at the more variable aspects of that porfolio, scale in terms of both dollars and disease burden. Research for platform technologies, those intentionally sought for application against a myriad of emergencies, receive an average of 12 million dollars annually. This is a very small fraction of awards: in 2016, this accounted for 1/5 of that spent on Zika, 1/10 that on Ebola, and 0.4% that on HIV. Although the NIH expends on what researchers request of it, scientists must submit proposals, and do so in ways that result in funding which includes preferentially choosing calls that are well funded. *Do the common elements of interceding or preventing epidemics from any pathogen or from classes of pathogen really amount to a percent or less of what must be known?*

A good example of consequences from planning based on pathogens rather than emergencies is plague. The pathogens that make the R&D Blueprint priority list cause substantial disease, may spread, disrupting public health infrastructures and economies. By those metrics, though, why are cholera and plague not on the list? With the possible exception of smallpox, the historical, acute devastation by plague in Europe and Asia has little rival. These emergencies continue today with Madagascar, India, and central Asian states having experienced recent outbreaks. A quick review of WHO Disease Outbreak News over the last 5 years reveals that plague-related news postings easily are in the top 10, if not in the top five of reported events.

### Why does plague not make the priority lists?

The common response from colleagues is that antibacterial drugs exist and can be easily distributed against plague, in particular fluoroquinolones such as ciprofloxacin, so plague should not be a priority. Ciprofloxacin has been around for years and yet we continue to have significant outbreaks of plague, recently a large one. A list and priority process performed completely differently, however, might ask what resources are needed to stop outbreaks rather than what pathogens need to be stopped. That list might have focused on the need for easily disseminated antibacterial therapies, as soon as they are needed. It might have sparked logistics or drug formulation research. Fights against pneumonic plague, such as those against MERS-CoV, pandemic influenza, and many other pathogens, would be helped by a listed need for better community-based infection, prevention, and control research, leading to measures that are practical, culturally acceptable, and fit for rapid field deployment. Many proactive risk management approaches would benefit from better research on interdicting animal to human crossover events.

A priority list based on necessary functions in the prevention and interruption of emergencies is completely different, and must yield different foci for research than pathogen-based processes that seek to incorporate broader concepts as an afterthought. There are some very specific research funding grant mechanisms that increasingly attempt to target needs that are non-pathogen based. The CEPI and the Defense Advanced Research Projects Agency both have launched calls for proposals that seek novel technology platforms for developing vaccines and other medical products faster, less than the 10- to 20-year horizon presently faced by product developers. The DARPA and BARDA also have funding calls for enhancing how people fight infections generally rather than how to fight a specific infection. These funding practices should be more broadly adopted. However, they are initiatives arising without a supportive discourse about the many actions that must be taken to prevent or mitigate a health emergency, rather than what must be on hand against a specific pathogen.

A modified approach for setting priorities could result in more funding stability, unlike what has been experienced recently. From 2013 to 2016, NIH awards for research on Ebola and related viruses increased 6-fold and funding for Zika virus went from zero to more than 60 million dollars. On the other hand, Nipah virus, Rift Valley Fever virus, and CCHF virus research awards fell 50–100% between 2013 and 2016, despite all three of them being listed in the WHO R&D Blueprint. Applicants for awards must follow the guidance for the grants. When these rapid changes occur, some laboratories must walk away from research progress against important threats to be eligible for continued funding, even when that progress can only be made over time in sustained work.

Finally, this change in strategy—redefining what makes a good list, and how it is used if at all—could have beneficial impacts on mitigating large, transformative infectious disease emergencies that traditional R&D programming finds difficult. Decades of R&D have curbed, but not quelled, HIV, malaria, TB, and cholera from their dominance in impacting vulnerable areas, nor has the impact of pediatric enteric and respiratory diseases diminished in import. These threats are pervasive, despite massive investments over decades toward their mitigation. Although details matter, one reason for their continued circulation may be that we have not looked more closely at those at risk; instead, we have focused too singularly on traditional product development pathways. Compounding this is that R&D goals and regulatory guidance are sometimes absent diverse and evolving perspectives.

## Moving forward.

No one can predict the future. We must ensure that as we prepare for public health emergencies caused by infectious disease threats, we focus on positively influencing prevention and response rather than too much on the tools themselves. We need a bit less attention on what makes specific infectious disease pathogens different, and more on how they are alike in the ways that they cause outbreaks and impact communities. We should emphasize the question of what we need to do, rather than what we need to have.

